# Inhibitory Effects of the Two Novel TSPO Ligands 2-Cl-MGV-1 and MGV-1 on LPS-induced Microglial Activation

**DOI:** 10.3390/cells8050486

**Published:** 2019-05-22

**Authors:** Sheelu Monga, Rafi Nagler, Rula Amara, Abraham Weizman, Moshe Gavish

**Affiliations:** 1 Ruth and Bruce Rappaport Faculty of Medicine, Technion- Israel Institute of Technology, Haifa 31096, Israel; sheelumonga@hotmail.com (S.M.); rafi.nagler@gmail.com (R.N.); rula_amara@yahoo.com (R.A.); 2 Sackler Faculty of Medicine, Tel Aviv University, Tel Aviv 6997801, Israel; weizmana@gmail.com; 3 Research Unit, Geha Mental Health Center and Felsenstein Medical Research Center, Petah Tikva 4910002, Israel

**Keywords:** translocator protein (TSPO), neuroinflammation, cytokines, microglial activation, BV-2 cell line, 2-Cl-MGV-1, MGV-1, NF-κB

## Abstract

The 18 kDa translocator protein (TSPO) ligands 2-Cl-MGV-1 and MGV-1 can attenuate cell death of astrocyte-like cells (U118MG) and induce differentiation of neuronal progenitor cells (PC-12). Lipopolysaccharide (LPS) is a bacterial membrane endotoxin that activates cellular inflammatory pathways by releasing pro-inflammatory molecules, including cytokines and chemokines. The aim of the present study was to assess the immuno-modulatory effect of TSPO ligands in activated microglial cells. We demonstrated that the TSPO ligands 2-Cl-MGV-1 and MGV-1 can prevent LPS-induced activation of microglia (BV-2 cell line). Co-treatment of LPS (100 ng/mL) with these TSPO ligands (final concentration- 25 µM) reduces significantly the LPS-induced release of interleukin-6 (IL-6) from 16.9-fold to 2.5-fold, IL-β from 8.3-fold to 1.6-fold, interferon-γ from 16.0-fold to 2.2-fold, and tumor necrosis factor-α from 16.4-fold to 1.8-fold. This anti-inflammatory activity seems to be achieved by inhibition of NF-κB p65 activation. Assessment of initiation of ROS generation and cell metabolism shows significant protective effects of these two novel TSPO ligands. The IL-10 and IL-13 levels were not affected by any of the TSPO ligands. Thus, it appears that the ligands suppress the LPS-induced activation of some inflammatory responses of microglia. Such immunomodulatory effects may be relevant to the pharmacotherapy of neuro-inflammatory diseases.

## 1. Introduction

18 kDa Translocator Protein (TSPO) is known to possess immunomodulatory effects [1]. In addition, TSPO plays a role in other biological functions such as: steroidogenesis, apoptosis, differentiation of neuronal progenitor cells, cholesterol transport, mitochondrial respiration, mitochondrial permeability transition pore opening, and cellular proliferation [2]. The activity of the TSPO can be modulated by its endogenous ligands diazepam binding inhibitor (DBI) and porphyrins [3,4]. Curcumin is known as a herbal TSPO ligand [5]. Previous studies have shown that the compounds 2-Cl-MGV-1 [2-(2-chlorophenyl) quinazolin-4-yl dimethylcarbamate] and MGV-1 [2-phenylquinazolin-4-yl dimethylcarbamate] (designed by our laboratory) have low affinity for the TSPO [6]. The affinity of both 2-Cl-MGV-1 and MGV-1 (Ki value) is 825 nM [6]. In addition, 2-Cl-MGV-1 and MGV-1 appear to have beneficial effects in prevention of cell death and inflammatory processes in cell culture studies as well as in animal studies [6,7,8,9]. In the present study, we assessed the impact of 2-Cl-MGV-1 and MGV-1 on in vitro cellular and molecular processes associated with inflammatory responses of microglia.

The microglial cells are part of the immune system of the central nervous system (CNS) [10]. When an infectious agent crosses the blood–brain barrier (BBB), microglial cells are activated and the initiation of inflammatory responses takes place [10,11].

Lipopolysaccharide (LPS) is an endotoxin, found in the outer membrane of gram-negative bacteria that elicits strong immune responses.

Microglia are generally recognized as the brain’s resident macrophages and are considered to be pivotal players in innate immune/inflammatory responses in multiple neurologic disorders [12]. Activated microglia can secrete a wide range of pro-inflammatory cytokines, such as Interleukin (IL)-1β, IL-6, tumor necrosis factor (TNF)-α, and interferon (IFN)-γ [13].

Microglia and astrocytes present various toll-like receptors (TLRs). Microglial TLR-4 is activated by LPS [14]. The activation of TLR-4 by LPS induces intracellular signaling cascades (e.g., NF-κB, MAPKs and JAK-STAT), which in turn upregulate downstream pro-inflammatory mediators, including inducible nitric oxide synthase (iNOS), nicotinamide adenine dinucleotide phosphate (NADPH) oxidase, cyclooxygenase 2 (COX-2), and subsequent release of pro-inflammatory cytokines (e.g., IL-1β, IL-6, and TNF-α), chemokines (e.g., MCP-1), nitric oxide (NO) and prostaglandins [15]. Exaggerated microglial response may lead to neuronal damage [16]. 

NF-κB is a ubiquitous transcription factor that plays a critical role in the cells of the immune system. It controls the expression of various cytokines and the major histocompatibility complex genes. In unstimulated cells, NF-κB is inactive in the cytoplasm, bound to B-cells inhibitor kappa (IκB) proteins. Activation of the NF-κB pathway induces phosphorylation of IκBs, targeting them for degradation through the ubiquitin-proteasome pathway. Free NF-κB can then enter the nucleus, where it regulates expression of its target genes. The cellular marker of NF-κB activation is expressed as NF-κB p65 induction [5].

Activation of macrophages by LPS, a TLR4 ligand, induces reactive oxygen species (ROS) generation both intracellularly and extracellularly via NADPH oxidase [17,18] with intracellular ROS serving a signaling function (e.g., potentiation of TNFα secretion) and extracellular ROS affecting adjacent cells (e.g., cytotoxicity) [18]. In particular, activation of the TSPO leads to ROS generation, including cardiolipin oxidation. The oxidation of cardiolipins is suggested to cause detachment of cytochrome *c* from cardiolipins as well as activation of the voltage dependent anion channel (VDAC) by ROS [19].

Cytokines mediate either pro-inflammatory or anti-inflammatory responses. For example, IL-1β and TNF-α accelerate inflammation, whereas IL-4 diminishes inflammatory signaling [12].

M1 macrophages have the unique ability to metabolize arginine to the “harmful” molecule NO, whereas M2 macrophages can metabolize arginine to the “repair” molecule ornithine [8]. This is where the terms M1 pathway, which is pro-inflammatory, and M2 pathway, which is anti-inflammatory were defined. The markers for M1 pathway are IL-1β, IL-6, TNF-α and IFN-γ whereas for M2 are IL-10 and IL-13. 

M2 pathway includes IL-4 and/or IL-13, immune complexes with TLRs, IL-1 receptor ligands, and IL-10. M2 macrophages produce ornithine and polyamines through the arginase pathway. For example, allergic asthma is characterized by the presence of high levels of IL-4 and IL-13, which can induce M2 polarization [20,21,22].

TSPO ligands can affect inflammatory processes [3,23]. The primary intracellular location of TSPO is the outer mitochondrial membrane [24]. Interestingly, TSPO and its ligands, including 2-Cl-MGV-1 and MGV-1, also appear to be involved in microglia activation, which may have therapeutic implications [9,18,25]. In addition, TSPO expression is upregulated in different pathological conditions such as brain ischemia, certain forms of epilepsy, glioma, and inflammatory peripheral neuropathy [26,27,28,29]. It appears that TSPO is also involved in neurodegenerative disorders such as Parkinson’s disease, Alzheimer’s disease, brain trauma, and other neurodegenerative diseases, which are associated with microglial activation [27,28,29,30].

In a recent study, we found that the novel TSPO ligands 2-Cl-MGV-1 and MGV-1 can attenuate the LPS-induced elevation in COX-2, iNOS and NO in BV-2 microglia cell line [9]. The aim of the present study was to assess the possible immuno-modulatory impact of these two TSPO ligands on the M1 and M2 pathways of inflammation in BV-2 cell line. To this end, we assessed the effects of these TSPO ligands on microglial pro-inflammatory cytokines, ROS generation, cell metabolism, and M2 pathway (M2 inflammatory markers) to show the possible specificity of the immuno-modulatory effects of the ligands. Additionally, in order to identify the cellular mechanism that is involved in the blockade of the M1 pathway of inflammation, we assessed the impact of TSPO ligands on NF-κB p65 (pS536) protein activation. We also assessed IL-10 and IL-13 levels in order to detect polarization effect of transition from M1 to M2.

## 2. Methods

### 2.1. BV-2 Cells

The in-vitro model of microglia was the BV-2 cell line, derived from raf/myc- immortalized murine neonatal microglia (provided by Professor Zvi Vogel from the Weizmann Institute of Science, Rehovot, Isreal). These cells are most frequently used as a substitute for primary microglia in pharmacological, phagocytotic and immunological studies, since LPS-activated BV-2 cells present a similar response pattern as that of primary microglia [31].

These murine BV-2 microglia cells were cultured at 37 °C in 5% CO_2_ and 90% relative humidity. The BV-2 cells were incubated in Dulbecco’s modified Eagle’s medium containing 4.5 g/l glucose, 1 mM L-glutamine and supplemented with 5% fetal bovine serum, penicillin (100 U/ml), and streptomycin (100 μg/mL) [32].

### 2.2. Lipopolysaccharide (LPS) Exposure

BV-2 cells were seeded in 100 mm × 20 mm plates. After 48 hours, the cells were exposed to 100 ng/mL LPS (Sigma-Aldrich, Rehovot, Israel) for 24 hours, as previously described [32]. Following LPS exposure, the cells and media were collected for the different assays mentioned below.

### 2.3. Treatment with the Novel TSPO Ligands

The synthesis of the ligands is described in our previous publication [6,9]. Treatment included: 24-hour treatment of BV-2 cells with LPS (100 ng/mL), with or without the two ligands- 2-Cl-MGV-1 and MGV-1 (final concentration 25 µM each), according to methods described previously [9]. These TSPO ligands have been compared with non-steroidal anti-inflammatory drug (NSAID) [diclofenac sodium (DS)] in order to test the effect of the agents at the same concentration. In the simultaneous treatment, BV-2 cells were exposed simultaneously LPS and the TSPO ligands for 24 hours, whereas in the post-treatment, cells were exposed to the LPS for 24 hours and then treated with TSPO ligands for the next 24 hours. 

### 2.4. Trypan Blue Exclusion Dye Assay

This assay includes cell counts by microscopy. Viable cells exclude the Trypan blue dye, while nonviable cells absorb the dye and appear blue. The assay was performed according to the manufacturer’s instructions (Sigma-Aldrich, Rehovot, Israel). Briefly, cells were scraped off, and were re-suspended in fresh medium and a 200 µL sample was collected for cell counting. Cells were stained with Trypan blue at a final concentration of 0.05%, i.e., the medium and dye were mixed in a volume ratio of 1:1 and the stained cells were counted by hemocytometer using an inverted microscope. This assay was performed for cell counting in order to seed a desired number of cells.

### 2.5. LDH Assay

The cytoplasmic enzyme LDH is released into the medium when the cell plasma membrane is damaged and is indicative of cell death. Cell cytotoxicity was detected using the Cytotoxicity Detection Kit (LDH) (Roche Pharmaceuticals, Mannheim, Germany) according to the manufacturer’s instructions. Briefly, cells were grown in 96 well plates for 72 hours with the various concentrations of ethanol (vehicle). For the assay, the culture supernatants were collected and incubated with the reaction mixture from the kit for 15 minutes at room temperature on a shaker. The amount of formazan formed is proportional to the amount of LDH released. The resulting color intensity is proportional to the number of damaged cells. Absorbance at 492 nm with reference at 690 nm was measured with the Spectrophotometer Zenyth 200 (Anthos, Biochrom Ltd., Cambridge, UK).

### 2.6. XTT Assay to Assess Cellular Metabolism

A cell proliferation-XTT based assay kit (Biological Industries, Beit HaEmek, Israel) was used, as described previously, to assay the metabolic activity of the cells as an indication of cell proliferation, cytotoxicity, and apoptosis [33]. The 2,3-bis [2-methoxy-4-nitro-5-sulphophenyl]-2*H*-tetrazolium-5-carboxyanilide inner salt (XTT) assay is based on reduction of XTT by mitochondrial dehydrogenases of viable cells yielding an orange formazan product. Absorbance at 490 nm with reference at 620 nm was measured with the Spectrophotometer Zenyth 200 (Anthos, Biochrom Ltd., Cambridge, UK).

### 2.7. Nitric Oxide Assay

BV-2 cells were seeded in 24 well plates and incubated for 72 hours in complete medium. Then the medium was aspirated, and the cells were washed with PBS and replaced with starvation medium. Starvation medium included Dulbecco’s modified Eagle’s medium (DMEM) containing 4.5 g/L glucose with 5% fetal bovine serum, penicillin (100 U/mL), and streptomycin (100 μg/mL) (Biological industries, Beit haemek, Israel) with 100 ng/mL of LPS and 25 µM (final concentration) of DS was applied. The vehicle control contained 1% ethanol in the starvation medium. LPS contained 100 ng/mL of LPS in vehicle. The comparison groups contained 100 ng/mL LPS with DS. NO production was determined by measuring the levels of the NO metabolite nitrite (NO_2_^−^) in the medium by using a colorimetric reaction with Griess reagent; prepared with 0.1 g of sulphanilamide and 0.01 g of NEDD (N-(1-Naphthyl) ethylenediamine) (Sigma, Rehovot, Israel) along with 274 µL 10 N HCl and 9.76 mL of DDW, applied according to the manufacturer’s instructions. Then, equal volumes of cell supernatants and Griess reagent were mixed (100 µL). 0.1 M sodium nitrate was used to build a calibration curve. Absorbance was measured at 540 nm after 15 minutes, with a Spectrophotometer Zenyth 200 (Anthos, Biochrom Ltd., Cambridge, UK).

### 2.8. Enzyme-Linked Immuno-Sorbent Assay (ELISA)

ELISA kits were used for the assessment of the concentrations of IL-1β (Peprotech Asia, Rehovot, Israel. Cat. No. 900-M47), TNF-α (ab208348), IFN-γ (ab100689), IL-6 (ab222503), IL-10 (ab108870), and IL-13 (ab219634) in the cell culture supernatant and NF-κB p65 (ab176647) in the cell lysates (Abcam, Zotal, Tel Aviv, Israel). The levels of all the detected cytokines were compared between LPS exposed cells with the TSPO ligands treatment simultaneously. All the substances provided in the kit were reconstituted, aliquoted, and stored at −20 °C, except streptavidin which was stored at 4 °C in dark.

For plate preparation and standards: Capture antibody was diluted with PBS to a concentration of 1.0 µg/mL. Plates were sealed immediately after adding 100 µL to each ELISA plate well (NUNC- Immuno plate, Thermo Fisher Scientific, Waltham, MA). Then the prepared plate was incubated overnight at room temperature. Wells were aspirated and washed 4 times with 300 µL per well of wash buffer. Blocking was done with 300 µL of blocking buffer. Standards were diluted from 2000 pg/mL to 0.0 pg/mL (depending on the kit limits) to obtain a concentration curve.

Cells were incubated at room temperature for at least 2 hours and the ELISA assessment was performed in quadrate. Then the plates were aspirated, washed 4 times, and the detection antibody was added after dilution. Streptavidin-HRP was put into the wells of the plate. Plates were incubated for 30 minutes at room temperature. Plates were subjected to aspiration, washed 4 times, and 3,3′,5,5′-Tetramethylbenzidine (TMB), a liquid substrate (Sigma-Aldrich, Rehovot, Israel), was added to the wells of the plate. Plates were incubated at room temperature for color development for 20 minutes. 100 μL of 1 N HCl was added as Stop Solution. Color development was monitored with an ELISA plate reader at 450 nm with the reference wavelength set at 620 nm.

### 2.9. Cardiolipin Content Assayed by Flow Cytometry Cell Sorting (FACS)

Cardiolipin in mitochondria contains a substantial proportion of highly unsaturated fatty acids and is therefore sensitive to oxidation. It is known that ROS can cause oxidation of cardiolipin. 10-N-Nonylacridine Orange (NAO) from Sigma-Aldrich (Rehovot, Israel) binds specifically to non-oxidized cardiolipin phospholipids. Thus, NAO fluorescence, measured with flow cytometry, was used to assay intact cardiolipin content in mitochondria, in order to determine the degree of oxidative injury to the mitochondria. For this assay, cells were seeded in 6-well plates. After 72 hours, LPS, with or without 2-Cl-MGV-1, MGV-1, and DS was applied. After being treated for 24 h, the cells were scraped off, collected from the wells, and centrifuged at 210× *g* for 5 min at 4 °C. Cell pellets were suspended in 0.5 mL of 10 μg/mL NAO in PBS and incubated for 30 min at 37 °C in the dark on a shaker. Then the cell suspensions were transferred to 5 mL FALCON FACS tubes and analyzed with BD FACS Calibur flow cytometer using CellQuest software (BD Biosciences, Franklin Lakes, NJ, USA).

### 2.10. Statistical Analysis

Results are presented as Mean ± standard deviation (SD). Two-tailed Wilcoxon, Student’s t and one-way analysis of variance (ANOVA) tests were used as appropriate, followed by Bonferroni’s post-hoc test. Statistical significance was defined by *p* < 0.05.

## 3. Results

### 3.1. The Impact of Vehicle (0–1% ethanol) on BV-2 Cell Viability

LDH assay was applied to assess the impact of ethanol as a vehicle on microglial BV-2 cell death. To this end, we used various concentrations of ethanol (0–1%). No toxic effects of ethanol on BV-2 cells were detected in this concentration range (Supplemental Figure S1). Thus, 1% ethanol was used as vehicle in all the experiments.

### 3.2. The Effect of TSPO Ligands on Microglial Activation

**Figure 1:**
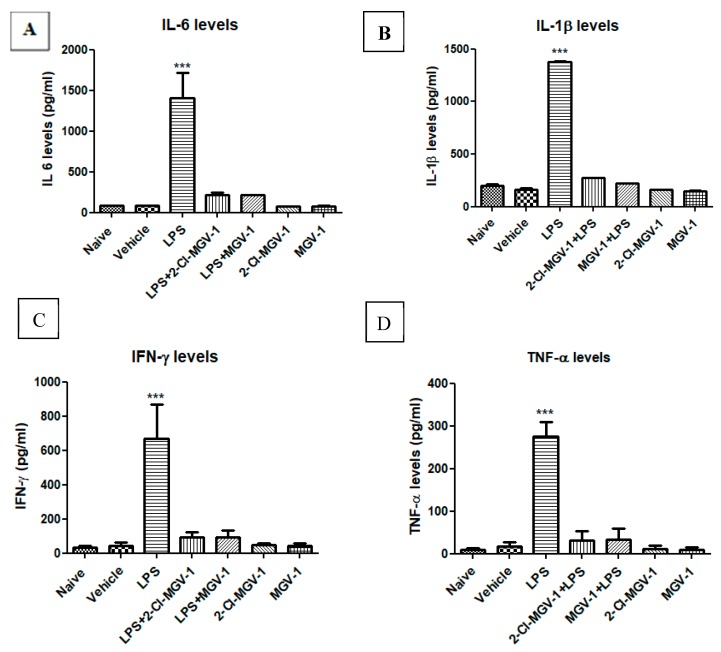
Effect of 2-Cl-MGV-1 and MGV-1 on the release of pro-inflammatory cytokines by Lipopolysaccharide (LPS)-induced activation of BV-2 cells. BV-2 cells were exposed to 100 ng/mL LPS for 24 hours with or without 2-Cl-MGV-1 and MGV-1 (final concentration 25 µM). (**A**) IL-6, (**B**) IL-1β, (**C**) IFN-γ, and (**D**) TNF-α levels (pg/mL for all). The levels of pro-inflammatory cytokines were calculated using a standard calibration curve and are presented as means ± SD; *n* = 4 per group in (A), (C), and (D); *n* = 6 in (B). ANOVA followed by Bonferroni’s post-hoc test was performed. *** *p* < 0.001 indicates significant differences of LPS treatment compared to all other treatment groups, including vehicle and naive. LPS+2-Cl-MGV-1 and LPS+MGV-1 did not differ significantly from both naïve and vehicle levels. All of these four cytokines are markers for M1 pathway of inflammation.

Exposure of BV-2 cells to LPS (100 ng/mL) resulted in a marked increase in the levels of the pro-inflammatory cytokines; (Figure 1A–D) as follows: induction in levels of IL-6 by 16.9-fold (Figure 1A), IL-1β by 8.3-fold (Figure 1B), IFN-γ by 16.0-fold (Figure 1C ), and TNF-α by 16.4-fold (Figure 1D) with respect to vehicle or naïve (ANOVA; F = 69.58, 2616, 33.8 and 101.7 respectively, *p* < 0.001 for all). As shown in Figure 1, treatment with both TSPO ligands 2-Cl-MGV-1 and MGV-1 (25 µM each) suppressed the LPS-induced release of the tested pro-inflammatory cytokines to control levels (Figure 1). The cytokine levels following TSPO ligand treatment along with LPS did not differ significantly from that of cells treated with vehicle alone (Figure 1A–D). Also, these TSPO ligands did not affect the levels of cytokines on their own (Figure 1A–D). ANOVA results remained statistically significant (*p* < 0.001) followed by Bonferroni’s post-hoc test. 

### 3.3. The Effect of TSPO Ligands on Microglial Cellular Metabolism

**Figure 2:**
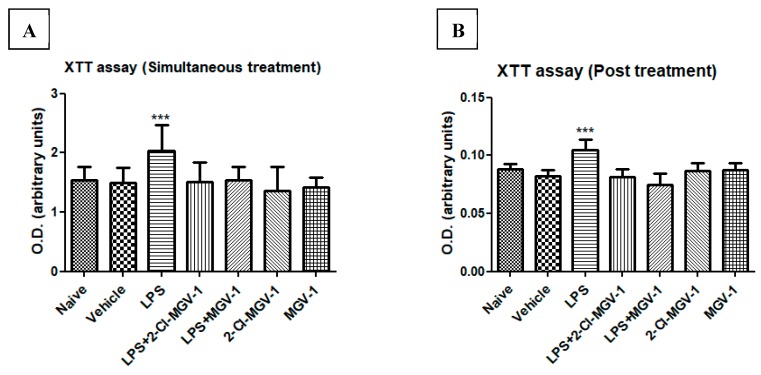
Cell metabolism with XTT assay of BV-2 cells. BV-2 cells were exposed to 100 ng/mL LPS for 24 hours (**A**) simultaneously with or without 2-Cl-MGV-1 and MGV-1, and (**B**) 2-Cl-MGV-1 and MGV-1 were added to BV-2 cells after 24 hours of LPS exposure (final concentration of ligands 25 µM each) for the next 24 hours. O.D. was recorded using an ELISA reader after XTT reaction and presented as means ± SD; *n* = 8. ANOVA followed by Bonferroni’s post-hoc test was performed, and the results remained statistically significant. *** *p* < 0.001 compared to all other groups.

We compared the effects of 2-Cl-MGV-1 and MGV-1 on LPS induced alterations in cellular metabolism. To this end, we used an XTT assay as an indicator of mitochondrial metabolic activity (as detailed in the Methods). No difference was observed between the naïve and vehicle control groups with regard to cellular metabolism. However, cellular metabolism in the LPS group was increased significantly by 26% with respect to vehicle. Treatment with each of the two ligands (25 µM each) simultaneously with LPS attenuated the metabolic response, and the values did not differ significantly from the naïve/vehicle levels (simultaneous treatment). These ligands alone did not affect the levels or intensity of cell metabolism (Figure 2A,B).

Cells were exposed to LPS for 24 hours and they exhibited increased metabolic activity (by 21%). After 24 hours, cells were treated with 2-Cl-MGV-1 and MGV-1 (25µM) for another 24 hours (post-treatment) and the metabolic activity was similar to that of the naïve/vehicle groups. The ligands did not show effects on their own. ANOVA followed by Bonferroni’s post-hoc test was performed, and the results remained statistically significant (*p* < 0.001 compared to naïve and vehicle).

### 3.4. Effect of 2-Cl-MGV-1 and MGV-1 on Cardiolipin Content

**Figure 3:**
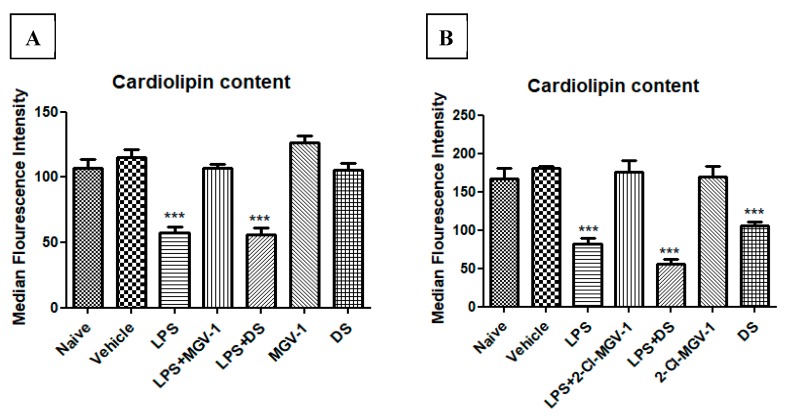
Effect of 2-Cl-MGV-1 and MGV-1 on the cardiolipin content (marker of ROS generation) in BV-2 cells: BV-2 cells were exposed to 100 ng/mL LPS for 24 hours with or without (**A**) MGV-1; (**B**) 2-Cl-MGV-1 with comparison to DS. Median Fluorescence Intensity (MFI) of cardiolipin content was calculated by FACS and presented as mean ±SD; *n* = 3 in each group. ANOVA followed by Bonferroni’s post hoc test was performed, and the results remained statistically significant (*** *p* < 0.001 compared to all other groups).

Decreased cellular cardiolipin content is a marker of ROS generation. BV-2 cells were exposed to 100 ng/mL LPS simultaneously with or without 2-Cl-MGV-1 or MGV-1 as compared to DS, which is an NSAID (final concentration 25 µM each). As shown in Figure 3A,B, the median fluorescence intensity of cardiolipin content, as measured by FACS, was decreased significantly in the LPS group by 45% as compared to naïve and vehicle groups. The levels of cardiolipin in LPS+2-Cl-MGV-1 and LPS+ MGV-1 groups were similar to those in naïve and vehicle groups and differed significantly from the LPS group alone. Also, 2-Cl-MGV-1 and MGV-1 did not affect cardiolipin levels on their own. DS was unable to reduce the ROS generation at a concentration of 25 µM; this experiment was performed in order to differentiate the anti-inflammatory property of the TSPO ligands from that of a typical NSAID, DS (Figure 3A,B). ANOVA followed by Bonferroni’s post hoc test was performed, and the results remained statistically significant (*p* < 0.001 compared to all other groups). 

### 3.5. The Effect of 2-Cl-MGV-1 and MGV-1 on NF-κB p65 (pS536)

**Figure 4:**
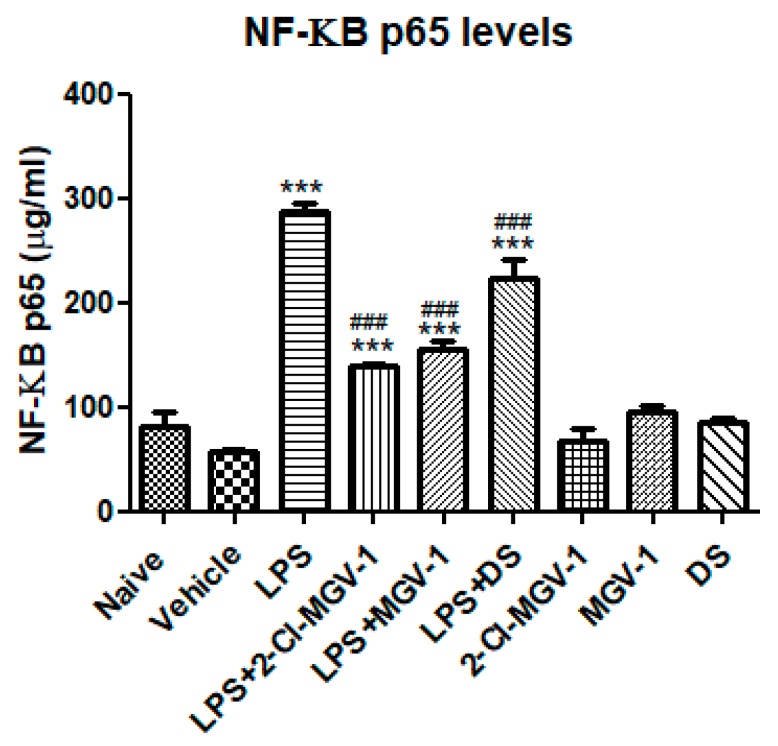
Effect of the two TSPO ligands on NF-κB p65 (pS536) levels in LPS- stimulated BV-2 cells. BV-2 cells were exposed to 100 ng/mL LPS for 24 hours with or without the two TSPO ligands or DS (25 µM each). NF-κB p65 (pS536) concentrations (µg/mL) were calculated using a standard calibration curve and are presented as means ± SD; *n* = 4 in each group. ANOVA followed by Bonferroni’s post-hoc test was performed, and the results remained statistically significant *** compared to all other groups and ### compared to LPS (*p* < 0.001 for all).

To gain insights into potential intracellular mechanisms involved in the inhibitory effects of 2-Cl-MGV-1 and MGV-1 on the M1 pathway of inflammation, we conducted an NF-κB p65 (pS536) ELISA assay in order to detect NF-κB p65 activation. As shown in Figure 4, exposure to LPS resulted in a 5-fold increase in NF-κB p65 as compared to vehicle whereas, just 2-fold increase occurred in the presence of the two TSPO ligands. Treatment with 2-Cl-MGV-1 and MGV-1 prevented the LPS-induced elevation the p65 levels. However, it remains significantly above the control levels. DS also exhibited significant inhibitory effect on LPS-induced p65 levels: 3.8-fold as compared to vehicle, 2.2-fold as compared to TSPO ligands and there is a difference of just 1.2-fold as compared to LPS.

### 3.6. Impact of the two TSPO Ligands on the M2 Pathway

**Figure 5:**
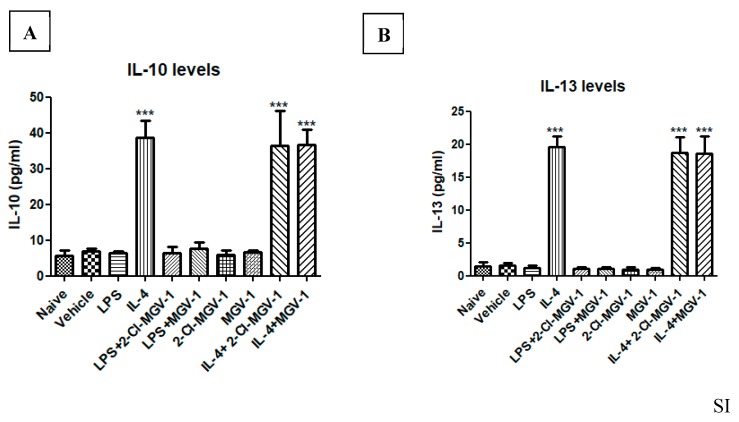
Effect of the two TSPO ligands on IL-10 and IL-13 levels in IL-4- stimulated BV-2 cells. (**A**, **B**) BV-2 cells were exposed to 10 ng/mL IL-4 for 24 hours with or without TSPO ligands (25 µM each). (**A**) IL-10 and (**B**) IL-13 concentrations (pg/mL) were calculated using a standard calibration curve and are presented as means ± SD; *n* = 4 in each group. ANOVA followed by Bonferroni’s post-hoc test was performed. *** *p* < 0.001 compared to all other groups. (IL-10 and IL-13 represent markers for the M2 pathway).

In an attempt to evaluate the possible impact of 2-Cl-MGV-1 and MGV-1 on the M2 pathway, we exposed BV-2 cells to IL-4. Exposure of the BV-2 cells to IL-4 was used as a positive control and resulted in the 4-fold increase in IL-10 and 23-fold increase in IL-13. The TSPO ligands did not affect the IL-4 induced stimulation of both IL-10 (Figure 5A) or IL-13 (Figure 5B), major markers for the M2 pathway. These results indicate that the suppression of M1 pathway by 2-Cl-MGV-1 and MGV-1 is not accompanied by a parallel effect on the M2 pathway. ANOVA followed by Bonferroni’s post hoc test was performed, and the results remained statistically significant (*p* < 0.001).

## 4. Discussion 

The major findings of this study are that the two novel TSPO ligands 2-Cl-MGV-1 and MGV-1 can attenuate the pro-inflammatory response of microglia to LPS stimulation. The anti-inflammatory effect of the ligands was revealed in the reduction of pro-inflammatory responses including increases in IL-1β, IL-6, TNF-α, IFN-γ, cell metabolism, and ROS initiation. This anti-inflammatory activity seems to be related at least to some extent to an inhibitory activity at the NF-κB, since both ligands attenuated LPS-induced stimulation of NF-κB p65 (pS536). However, it is of note that suppression of NF-κB p65 (pS536) was inhibited to an extent of almost 50%, while the release of the pro-inflammatory cytokines was inhibited almost completely. Thus, it seems that the TSPO ligands should have also an inhibitory impact on other inflammatory pathways that are independent of their effect on NF-κB. At the mechanistic level, it is possible that translocation of the TSPO into the cell nucleus or translocation of other proteins are also involved in the anti-neuroinflammatory effect of the TSPO ligands. Future studies are needed to identify such TSPO-sensitive inflammatory pathways. Unfortunately, the impact of the TSPO ligands on in vivo model of neuroinflammation are unavailable at present. Nevertheless, our study may be relevant to the treatment of neurological diseases suggested to be associated with neuroinflammation such as Parkinson disease, Alzheimer’s disease, Huntington disease, and traumatic brain injury. In addition, since neuroinflammation may also be involved in neuropsychiatric disorders like schizophrenia, major depressive disorder, and bipolar disorder, it is possible that the TSPO ligands may have a therapeutic role also in these disorders. Moreover, the anti-inflammatory activity was larger than that achieved by DS, a prototype of NSAID. The anti-inflammatory effect of the ligands was restricted to M1 pathway. These results are consistent with our recent study that showed a suppressive effect of the ligands on two other inflammatory intracellular markers namely COX-2 and iNOS [9]. 

Microglia, the resident immune cells of the central nervous system, receive signals from various stimuli ranging from pathogenic invasions, stress, toxins and autoimmune processes which results in neurodegenerative processes. Such signals indicate disruption of normal cellular functioning and lead to the activation of microglia [34]. Activated microglia release the endogenous inflammatory factors to activate other cells in the nearby vicinity, thus proceed to evoke acute or chronic inflammation. Microglial activation is the hallmark of neuroinflammation in several neurodegenerative diseases and pathological conditions of the CNS [35]. Inflammatory response in the CNS is mediated by microglial activation [36]. Microglia activation states may be treated by attenuation of M1 responses or promotion of M2 responses [37]. Activation of microglia produces a plethora of inflammatory mediators including IFN-γ, IL-1β, IL-4, IL-6, IL-10, IL-12, IL-13, TNF-α, metalloproteinases, NO, and ROS [14,38]. It is known that ROS decreases the membranal cardiolipin content. Our TSPO ligands inhibited the LPS-induced reduction in cardiolipin content, an activity that reflects prevention of ROS production. However, these TSPO ligands did not affect IL-4 induction of IL-10 and IL-13 (markers of M2 pathway). In a previous study with macrophages, it was shown that LPS can cause induction of IL-10 [39]. We used the BV-2 microglial cell line as a model and we did not assess the impact of LPS on IL-10 release in our microglial cells. It is unclear whether microglial cells differ from macrophages in their response to LPS, with regard to IL-10 or IL-13 release. We focused in the present study on the potential inhibitory effects of the TSPO ligands on the M1 pro-inflammatory pathway. LPS is known to induce M1 pro-inflammatory pathway [40], while IL-10 is a marker of the M2 anti-inflammatory pathway [41]. Since the TSPO ligands did not affect the induction of IL-10 and Il-13 by IL-4, it is unlikely that they have a significant modulatory effect on the M2 anti-inflammatory pathway, and it is likely that they are inhibitors of the M1, but not the M2, pathway. Inhibiting the M1 phenotype while stimulating the M2 phenotype has been suggested as a potential strategy for the treatment of neuroinflammatory disorders [42]. Unfortunately, most of the compounds reducing neuroinflammation suppress M1 phenotype, while only few compounds have been demonstrated to promote the polarization to the M2 phenotype [43,44,45]. LPS is a classical TLR4 agonist that polarizes microglia into the M1 pro-inflammatory phenotype and also inhibits the expression of M2-related anti-inflammatory markers, leading to induction of inflammatory responses [46,47]. The primary immune effector cells of the CNS, microglia respond to injury or to the presence of pathogens by becoming activated (M1 pathway). Upon activation, microglia undergo proliferation, chemotaxis, and morphological alterations and generate numerous mediators involved in inflammatory and immunomodulatory responses [15]. Our ligands were able to attenuate some of the LPS-induced inflammatory processes.

According to our previous studies, 2-Cl-MGV-1 and MGV-1 can attenuate cell death in astrocytes like U118MG cells and can induce differentiation in neuron-like PC-12 cells as well [6]. A recent study shows that 2-Cl-MGV-1 and MGV-1 can suppress the LPS-induced accumulation of NO in BV-2 cells [9]. Moreover, these two ligands were found to be potent anti-inflammatory agents, in contrast to the weak effect of DS.

Furthermore, these ligands block the M1 pathway without polarizing the phenotype into M2. The lack of activation of M2 phenotype may be an advantage since stimulation of this pathway is associated with hypersensitivity, allergy, and tumor progression [48,49].

We have found in the current study that the TSPO ligands 2-Cl-MGV-1 and MGV-1 can reduce the LPS-induced microglial generation of NO, IL-1β, IFN-γ, and TNF-α. In this context, IL-1β elevation can lead to apoptosis, and together with TNF-α may cause damage to the blood–brain barrier and infiltration of leukocytes [50]. We have shown the anti-inflammatory activity of 2-Cl-MGV-1 and MGV-1 is may be related to a suppressive effect on NF-κB p65 activation. NF-κB plays a pivotal role in immune cells and is rapidly activated by a wide variety of pathogenic signals and functions as a potent and pleiotropic transcriptional activator [51]. Attenuation of the NF-κB activation may be beneficial in suppressing toxic/septic shock, graft vs. host reactions, some acute inflammatory reactions, and acute-phase response [5]. The LPS-induced elevation of IL-1β release could be related to NF-κB activation, since activated NF-κB migrates to the nucleus, where it induces the transcription of inflammatory genes, like IL-1β [52]. So, it seems that the inhibition of NF-κB activation is involved in the anti-inflammatory effects of the TSPO ligands. Several pro-inflammatory mediators including IL-1β, iNOS, IFN-γ, IL-6, IL-23, and TNF-α are secreted by activated microglia [53]. Our present results are consistent with our recent study that demonstrated that these TSPO ligands can counteract with iNOS, COX-2, and NO in LPS-induced activation of microglia [9].

In conclusion, our current study shows that the TSPO ligands 2-Cl-MGV-1 and MGV-1 are capable to suppress the inflammatory responses associated with LPS-induced activation of microglia. This potent anti-inflammatory activity may be relevant to the treatment of neuroinflammatory diseases. The anti-inflammatory activity achieved by the two TSPO ligands was much stronger than that achieved by the NSAID, DS. It is of note that the magnitudes of the anti-inflammatory activities of 2-Cl-MGV-1 and MGV-1 were very similar without any significant differences in the impact of these two ligands on pro-inflammatory mediators. The relevance of the findings in microglial cells to peripheral inflammatory processes and diseases merits further investigation.

## Supplementary Materials

The following are available online at https://www.mdpi.com/2073-4409/8/5/486/s1.
