# Supplementary material for: Inhibitory Effects of the Two Novel TSPO Ligands 2-Cl-MGV-1 and MGV-1 on LPS-induced Microglial Activation
Source: Cells. 2019 May 22;8(5):486. doi: 10.3390/cells8050486 (PMC6562711; doi:10.3390/cells8050486)
Supplement: Supplementary file 1 [file cells-08-00486-s001.pdf]

Supplementary data

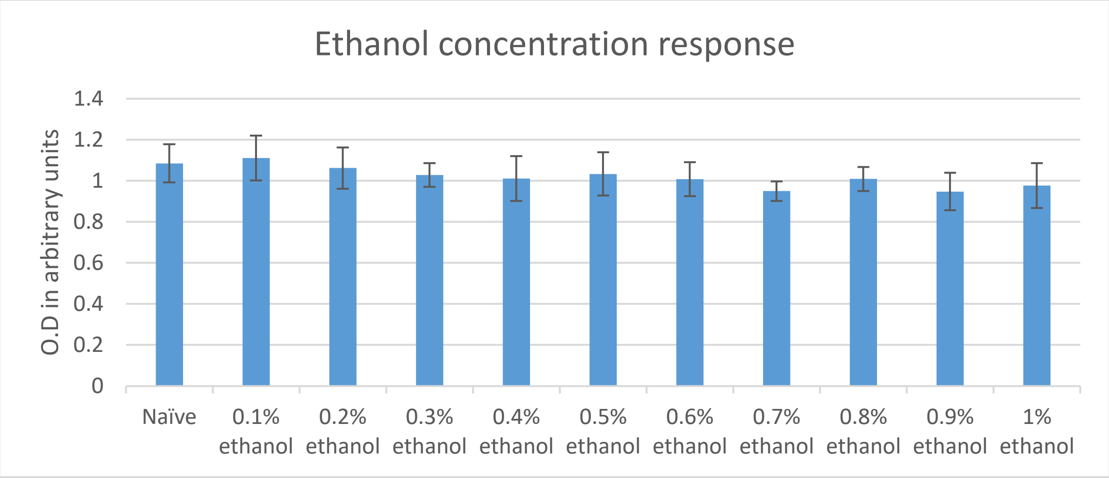

Figure S1. Ethanol concentration response on BV-2 cell death as measured by LDH release. BV-2 cells (n = 8 in each group) were exposed to various concentrations of ethanol (0% to 1%) for 24 hours and LDH assessment was performed. No significant changes in LDH levels for the various concentrations of ethanol were detected. (ANOVA, $p > 0.05$).

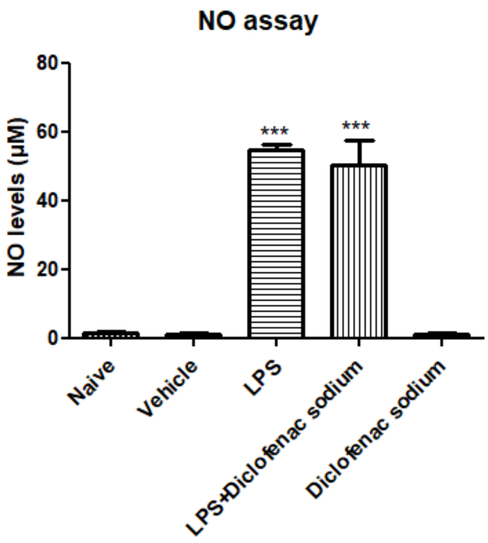

Figure S2. Effect of diclofenac sodium on nitric oxide levels in BV-2 cells. BV-2 cells were exposed to 100 ng/ml LPS for 24 hours simultaneously with or without diclofenac sodium (25 μM). NO levels were calculated using a standard calibration curve and presented as means ± SD; n = 8. ANOVA with Bonferroni's post-hoc test was performed. *** $p < 0.0001$ compared to both naïve and vehicle groups.
